# Drug resistance–associated mutations in *Plasmodium* UBP-1 disrupt its essential deubiquitinating activity

**DOI:** 10.1016/j.jbc.2025.108266

**Published:** 2025-02-03

**Authors:** Cameron J. Smith, Heledd Eavis, Carla Briggs, Ryan Henrici, Maryia Karpiyevich, Megan R. Ansbro, Johanna Hoshizaki, Gerbrand J. van der Heden van Noort, David B. Ascher, Colin J. Sutherland, Marcus C.S. Lee, Katerina Artavanis-Tsakonas

**Affiliations:** 1 Department of Pathology, University of Cambridge, Cambridge, UK; 2 Department of Immunology, London School of Hygiene & Tropical Medicine, London, UK; 3 Wellcome Sanger Institute, Wellcome Genome Campus, Hinxton, UK; 4 Leiden University Medical Center, Leiden, Netherlands; 5 University of Queensland, School of Chemistry and Molecular Biosciences, Queensland, Australia; 6 Biological Chemistry and Drug Discovery, Wellcome Centre for Anti-Infectives Research, University of Dundee, Dundee, United Kingdom

**Keywords:** *Plasmodium*, DUB, ubiquitin, parasite, ubiquitin hydrolase

## Abstract

Deubiquitinating enzymes function to cleave ubiquitin (Ub) moieties from modified proteins, serving to maintain the pool of free Ub in the cell while simultaneously impacting the fate and function of a target protein. Like all eukaryotes, *Plasmodium* parasites rely on the dynamic addition and removal of Ub for their own growth and survival. While humans possess around 100 deubiquitinases, *Plasmodium* contains ∼20 putative Ub hydrolases, many of which bear little to no resemblance to those of other organisms. In this study, we characterize *Plasmodium falciparum* UBP-1, a large Ub hydrolase unique to *Plasmodium* spp., which has been linked to endocytosis and drug resistance. We demonstrate its Ub activity, linkage specificity, and assess the repercussions of point mutations associated with drug resistance on catalytic activity and parasite fitness. We confirm that the deubiquitinating activity of UBP-1 is essential for parasite survival, implicating an important role for Ub signaling in endocytosis.

*Plasmodium*, like all other eukaryotes, deploys post-translational protein modifications as a means of maintaining homeostasis while driving the progression of its life cycle. Components of the ubiquitin (Ub) pathway mediate certain critical cellular processes, including protein degradation and trafficking, and are necessary to parasite survival (1). The temporal and functional characterization of Ub pathway enzymes during *Plasmodium* development may uncover novel drug targets with the potential to provide new and urgently needed treatments for malaria.

Ub is attached to protein substrates *via* an enzymatic cascade consisting of an ATP-dependent E1-activating enzyme, an E2-conjugating enzyme, and an E3 ligase. This modification is dynamic and can be reversed through the action of Ub hydrolases (deubiquitinases [DUBs]). *Plasmodium falciparum* expresses around 20 putative DUBs, annotated by homology with other eukaryotic enzymes, but the complexity and function of the malarial Ub system remains mostly uncharacterized. UBP-1, a putative *Plasmodium* Ub hydrolase, was first highlighted in *Plasmodium chabaudi* and linked to drug resistance (2). By sequencing *P. chabaudi* clones evolved *in vivo* in response to artesunate and chloroquine treatment, two separate valine to phenylalanine (Val to Phe) point mutations were identified in resistant parasites that both occurred within the C-terminal region of UBP-1. Two subsequent studies extended this link to *Plasmodium berghei* (3) and *P. falciparum* (4) through transgenic introduction of the corresponding point mutations into wildtype lines, demonstrating that modified parasites had increased resistance to artemisinin and in the case of *P. berghei* to chloroquine as well. Moreover, disruption of UBP-1 protein localization through knock-sideways technology in lab-adapted *P. falciparum* also confers artemisinin resistance (5). Although the Val to Phe mutations have not been identified in field isolates, other naturally occurring replacements within the *Pf*UBP-1 (*Plasmodium falciparum* UBP-1) sequence, such as R3138H, have been correlated with artemisinin resistance (6).

The UBP-1 gene locus is located on chromosome 1 and consists of three exons encoding a large protein of 416 kDa. Although other *Plasmodium* species all appear to contain a UBP-1 homolog, higher eukaryotes lack homologs, barring low-identity hits to the catalytic domain alone. Transcriptomic analysis shows that it is predominantly transcribed in merozoites and rings (7), suggestive of a role in these early stages of infection. Indeed, UBP-1 appears to regulate the process of endocytosis, critical for the growth and maturation of the blood stages. It localizes to the parasite cytostome, the site of initiation for uptake of host cell cytosol, and in parasites where UBP-1 has been perturbed, food vacuoles appear significantly smaller (5). The parasiticidal activity of artemisinin is dependent on efficient uptake and transfer of hemoglobin to the food vacuole during early ring stages of development. Once there, artemisinin is transformed to its active form by interacting with heme (8). Thus, impaired endocytosis would result in lower uptake of hemoglobin and link UBP-1 function to artemisinin resistance. Together, these findings all point to a critical role for this protein in mediating drug susceptibility.

As the V3275F and V3306F mutations related to *in vitro* drug resistance occur near or within the UCH domain of UBP-1, we sought to explore its putative function as a Ub hydrolase and to assess the effects of these mutations on catalytic function. Knock-sideways mutagenesis suggests that *Pf*UBP-1 is essential for parasite viability (5). However, disruption of the entire gene does not pinpoint the specific function or region of this protein that is required: is it the DUB activity or is it something related to the large upstream portion of *Pf*UBP-1? In this study, we aimed to characterize the Ub hydrolase activity of *Pf*UBP-1, to assess the repercussions of these point mutations on catalytic activity and parasite fitness, and to gain insight into the function of this essential protein. We validate *Pf*UBP-1 as a true deubiquitinating enzyme with wide specificity for a variety of Ub linkages. We further demonstrate that although V3275F and V3306F dramatically reduce catalytic activity, they leave parasite fitness unaffected, whereas the complete ablation of catalytic activity *via* a C3179A mutation is lethal. Our findings raise interesting questions about the putative function of UBP-1 and its role in mediating both endocytosis and artemisinin resistance.

## Results

### *Pf*UBP-1 V to F mutations affect DUB activity but not fitness

**Figure 1:**
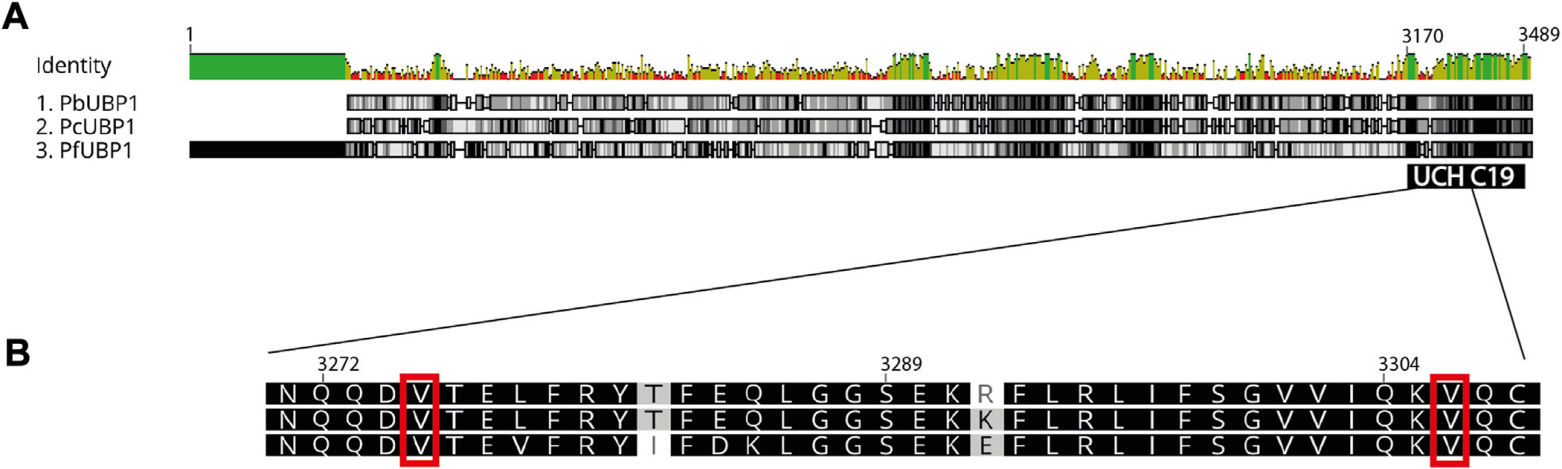
***Plasmodium* spp. UBP-1 alignment.***A*, alignment of the full sequence of UBP-1 from *Plasmodium berghei* (*Pb*), *Plasmodium chabaudi* (*Pc*), and *Plasmodium falciparum* (*Pf*) showing high (*green*) and low (*red*) identity regions and conserved amino acids in grayscale. *B*, detail of alignment of the UCH domain portion containing the two valine residues mutated in this study.

*Pf*UBP-1 is a large, 3753 amino acid protein (Fig. 1*A*) with a predicted UCH C19 peptidase domain at its C terminus and no other recognizable motifs. Although overall sequence identity between UBP-1 orthologs of different *Plasmodium* species is relatively low, the UCH domain and particularly the Val residues associated with drug resistance are conserved (Fig. 1*B*). The modeled structure of the predicted C19 peptidase domain aligned closely with experimental structures of Ub carboxyl-terminal hydrolases (sequence identities up to 33%, rmsd <1 Å across residues 3181–3489), consistent with its proposed function; however, the upstream region was predicted to be largely disordered and of low confidence.

**Figure 2:**
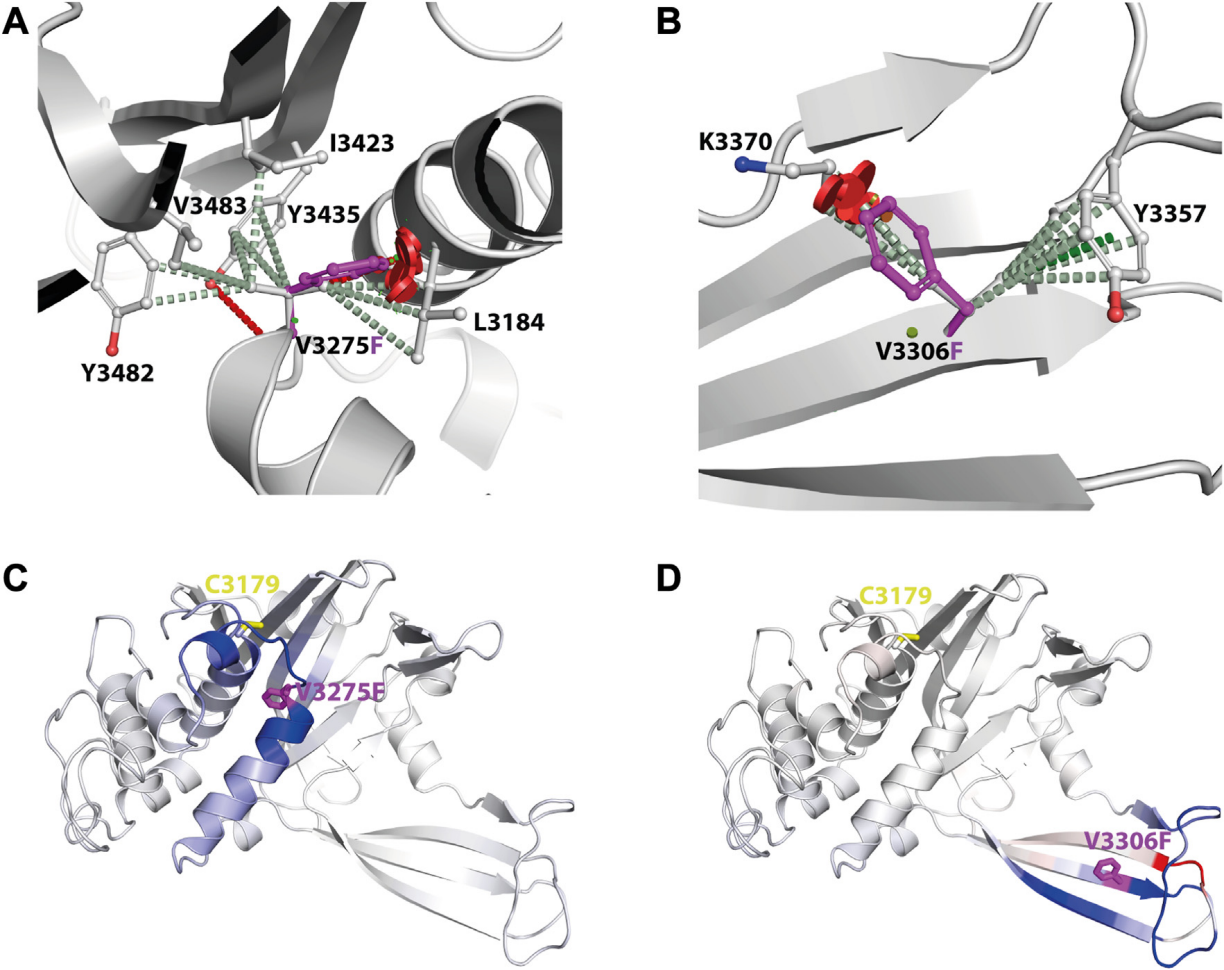
**Molecular depiction of the effects on the *Pf*UBP-1 UCH domain caused by mutations V3275F and V3306F**. The molecular interactions made by V3275 (*A*) and V3306F (*B*) were calculated and shown by Arpeggio as *dashed lines*. Hydrogen bonds are shown as *red dashed lines*, hydrophobic as *light green*, and pi as *deep green*. Steric clashes caused by the mutations to phenylalanine are shown as *red disks*. The change in vibrational entropy energy, calculated by DynaMut, from the wildtype to V3275F (*C*) and V3306F (*D*), where residues are colored from *blue*, representing a rigidification of the structure, to *red*, a gain in flexibility. PfUBP-1, Plasmodium falciparum UBP-1.

As both Val residues that are mutated in the drug-resistant parasites lie within the C19 peptidase domain, we hypothesized that introducing a Phe at either position would result in protein misfolding and/or impair catalytic activity. Both V3275 and V3306 are buried and proximal to the active site cysteine, and their mutation to Phe would result in a large change in overall residue volume within the tightly packed protein core. Modeling the mutations suggests that a change in either position would result in significant steric clashes (Fig. 2, *A* and *B*), altering the local protein structure and potentially disrupting the active site architecture *via* changes to molecular packing and flexibility of the surrounding region. This was consistent with predictions that both mutations would lead to a significant decrease in protein stability (∼kcal/mol), by SDM2, mCSM-Stability, DUET, and DynaMut. Normal mode analysis indicated that both mutations would lead to altered protein dynamics and flexibility, which can be visualized as changes in the vibrational energy (Fig. 2, *C* and *D*). The propagation of these changes was predicted to alter the dynamics near the active site cysteine.

**Figure 3:**
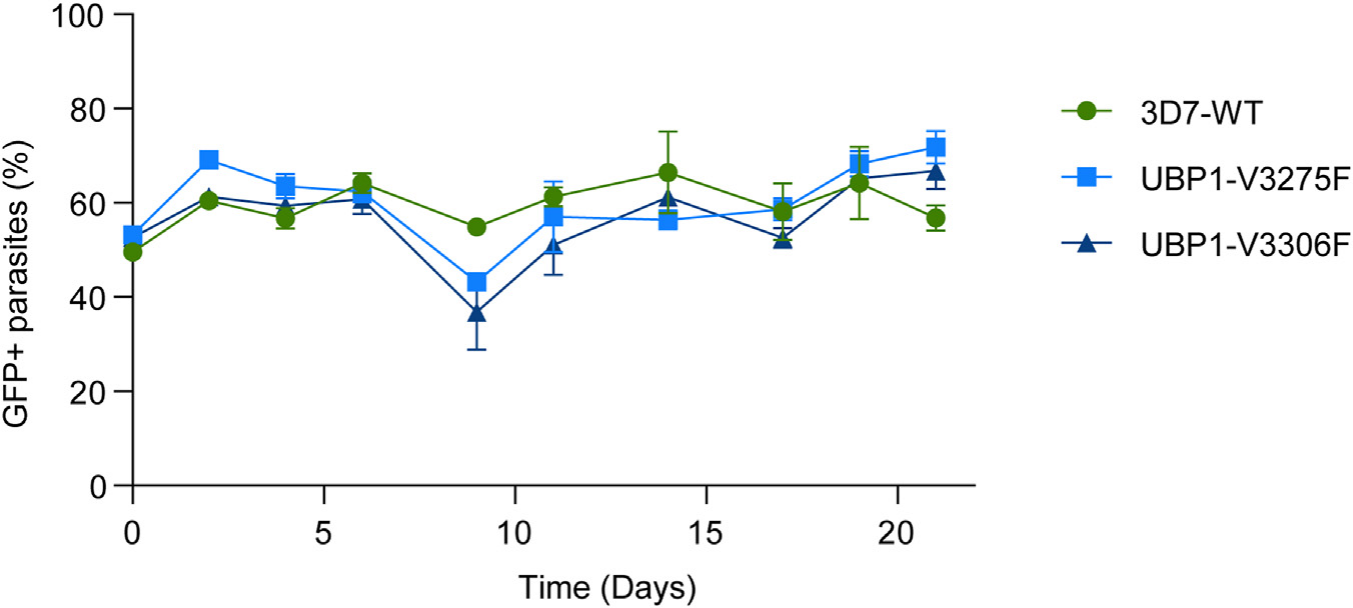
**Fitness of UBP-1 V3275F and V3306F mutant parasites.** UBP-1 mutant lines V3275F and V3306F or wildtype 3D7 were individually competed against a fluorescent reporter line Dd2-EGFP. Parasites were mixed at a 1:1 ratio, and the relative proportions of nonfluorescent test *versus* fluorescent reference line were measured by flow cytometry at regular intervals, with total parasites detected by staining with MitoTracker Deep Red. Shown is the average of three independent cultures, with error bars representing standard deviation.

As V3275F and V3306F transgenic parasites have been shown to be viable (4), we used these lines in a head-to-head competition assay to determine if our hypothesized impairment in DUB activity would result in more subtle differences in growth rates. V3275F, V3306F, and the parental 3D7 line were each cocultured at a 1:1 ratio with a fluorescent reference line. Over the course of 3 weeks and at regular intervals, mixtures were stained with MitoTracker Deep Red, and the ratio of GFP positive to negative parasites was detected by flow cytometry to measure relative growth. Results showed equivalent rates of growth for both mutant lines as compared with the parental 3D7 (Fig. 3) and, notably, no measurable reduction in fitness for either UCH-domain mutant.

### *Pf*UBP-1 contains a functional UCH domain

In light of these results, we questioned whether the DUB domain is indeed functional. To directly demonstrate deubiquitinating activity of UBP-1, we generated a truncation mutant of the UCH domain and used a bacterial expression system to produce recombinant protein. The truncation was designed to encompass as little extra sequence as possible outside the UCH domain while preserving alpha helices and structural integrity. Obtaining a measurable amount of protein was not trivial, as the entirety of UBP-1 is too large for bacterial expression, and many truncations failed to express altogether. Several different combinations of truncations and bacterial strains were tested as well as an extensive array of expression, lysis, purification, and buffer conditions. Ultimately, using low IPTG levels for induction and expressing the proteins at 18 °C, we were able to express a fragment containing the last 548 amino acids of UBP-1 with a molecular weight of 64.5 KDa (UBPtrunc) and through site-directed mutagenesis introduced the V3275F and V3306F mutations. We also mutated the active site Cys3179 to alanine to assess whether proteolytic function is dependent on the canonical Cys-His-Asp triad of the UCH domain. Protein levels were too low to observe by Coomassie gel staining (Fig. S2*B*); however, bands of the correct size were observed by Western blot (Fig. S2*A*). As elution of the protein from His-beads resulted in a loss of activity, assays were performed with the protein on-bead, with concentrations normalized by Western blot.

**Figure 4:**
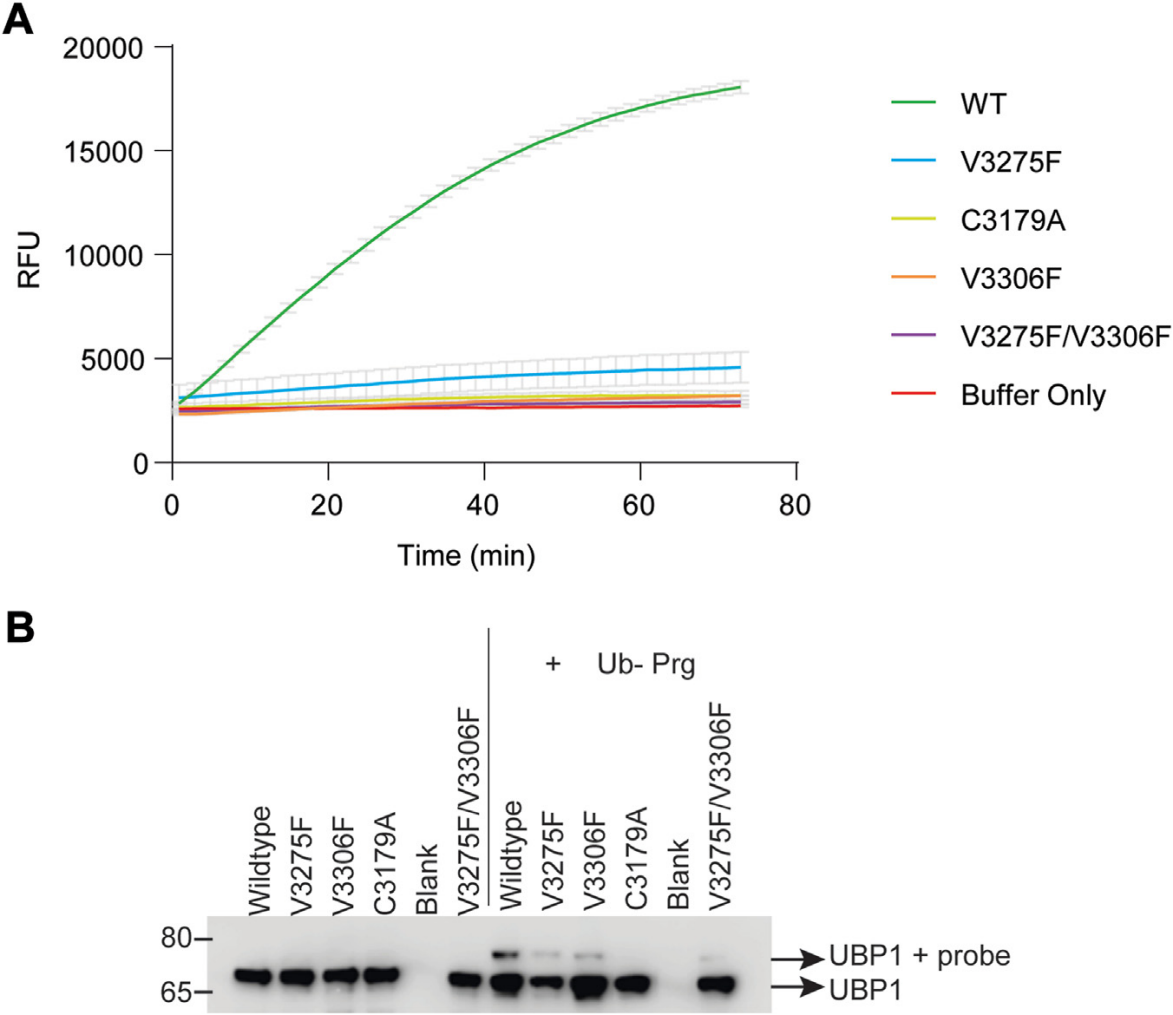
**DUB activity of *Pf*UBP-1 wildtype and V3275F and V3306F mutant proteins.** The *Pf*UBP-1 C-terminal truncation (2952-3499) was expressed in wildtype, V3275F, V3306F, C3179A, and V3275F/V3306F forms with a 6xHIS tag. *A*, proteins were reacted with Ub–AMC substrate over the course of 75 min (*x*-axis), with release of fluorescence (measured in relative fluorescence units, *y*-axis) indicative of activity. Constructs in the legend are ranked according to their final measurements in the assay, for clarity. Reactions were done in triplicate. *B*, proteins were also reacted with a Ub–Prg probe. A shift up in electrophoretic mobility detected by anti-His immunoblot represents binding of the probe and enzymatic activity. An image of the uncropped gel can be found in Fig. S2*A*. DUB, deubiquitinase; *Pf*UBP-1, *Plasmodium falciparum* UBP-1; Ub–AMC; Ub–Prg, ubiquitin–amido-methyl-coumarin.

Ub hydrolysis of UBPtrunc was first tested using a Ub–AMC (Ub–amido-methyl-coumarin) fluorescence intensity assay, which relies on real-time measurement of AMC fluorophore release from the Ub reagent by the enzyme. In this assay, enzymes perform multiple catalytic cycles until all the Ub–AMC substrate is depleted, providing information on activity kinetics and allowing direct comparisons between mutants. The wildtype construct generated a robust activity curve, which flattened once substrate was depleted, whereas the mutant proteins failed to demonstrate any appreciable activity above baseline and behaved similarly to the C3179A negative control and the buffer-only sample (Fig. 4*A*).

To further investigate the mode of action of UBPtrunc and the effect of the mutations on the catalytic mechanism, we reacted wildtype and mutant proteins with HA-Ub–Prg (ubiquitin–propargylamide), a Ub-derivatized probe designed to covalently bind the highly nucleophilic active site cysteine of deubiquitinating enzymes (9). An active Ub hydrolase would be expected to form a covalent and noncleavable complex with the probe and thus migrate at a higher molecular weight in SDS-PAGE analysis, as indeed is observed when reacting wildtype PfUBPtrunc with the probe (Fig. 4, lane 7). The C3179A mutation rendered the UCH completely inactive as no complex formation was observed (lane 10). Both V3275F and V3306F mutants showed impaired complex formation by gel-shift assay, and the double mutant showed an even more pronounced effect on complex formation, although in all V-to-F mutants, some minor residual activity did remain (Fig. 4*B*). Of note, this type of assay results in the active site cysteine being covalently coupled to the Ub-based probe, and hence, each UBP-1 enzyme can only react once with the suicide probe.

Together, these results demonstrate that UBP-1 does contain a functional UCH domain but also that the V to F mutations do impair proteolytic activity as predicted by our models. The lack of activity for the C3179A catalytically dead mutant across both assays indicates that the observed activity for the wildtype protein is not because of background contamination. A closer look at these predicted structures in Figure 2 suggests two ways these mutations may affect the UBP-1 interaction with Ub. In the case of V3275F, the additional steric bulk of the Phe within the small binding groove effectively narrows the access to the enzyme’s active site, which may partially block Ub binding. On the other hand, V3306 seems to form part of a Ub-binding S1 pocket interface. Thus, the Val to Phe mutation at this position may disrupt the interaction between Ub and UBP-1 and thus the orientation necessary to allow catalysis.

#### UBP-1 deubiquitinating activity is essential for parasite survival

As no growth defect was observed for the V3275F and V3306F mutant parasites despite their minimal activity in our *in vitro* assays, we wanted to ascertain whether the deubiquitinating activity of UBP-1 is essential to the parasite. We initially attempted to generate C3179A parasites using CRISPR–Cas9 but were unsuccessful in three attempts, whereas the control C3179C plasmid using the same guide was successful. Interestingly, one transfection generated a parasite line in which the guide sequence was mutated (shield mutations) but not the active cysteine (Fig. S1). This suggests the parasites used a hybrid of the endogenous sequence and the repair plasmid to repair the double-strand break.

**Figure 5:**
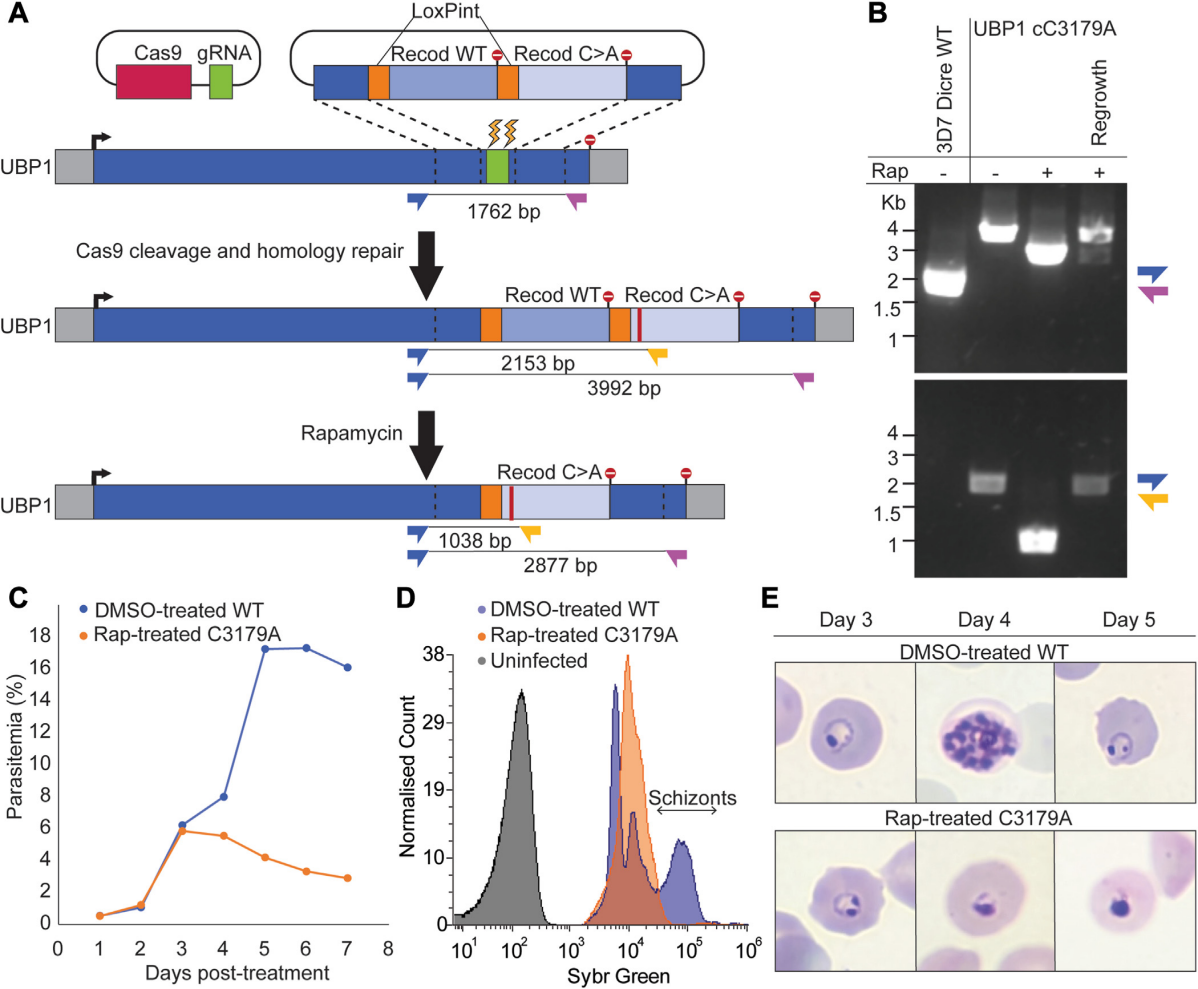
**Effect of a *Pf*UBP-1 C3179A mutation on parasite fitness.***A*, schematic depiction of the UBP-1 conditional mutation strategy. *B*, PCR validation of UBP-1 modification. *Arrows* on the schematic shown in *A* represent primers used for PCR, with expected band sizes depicted. *C*, growth curve of DMSO *versus* Rap-treated parasites. Parasites were Rap-treated at day 0, and parasitemia was measured by flow cytometry daily for 7 days. *D*, flow cytometry histogram of SYBR Green–stained untreated and Rap-treated parasites on day 4 post-treatment. Schizonts contain high DNA levels. *E*, Giemsa smears of DMSO and Rap-treated C3179A parasites at days 3, 4, and 5 post-treatment. DMSO, dimethyl sulfoxide; *Pf*UBP-1, *Plasmodium falciparum* UBP-1; Rap, rapamycin.

To further investigate the essentiality of the deubiquitinating activity, we generated a conditional mutant parasite line, where the active site cysteine would be replaced by alanine upon treatment with rapamycin (Rap). A recodonized version of the wildtype UCH domain was flanked by loxP sites within a synthetic intron, followed by an alternatively recodonized UCH domain in which the active site cysteine was replaced with alanine (Fig. 5*A*). Addition of Rap activates the DiCre recombinase within the 3D7 DiCre line, inducing excision of the wildtype domain and moving the mutant domain in frame with the N terminus of the gene. Modification of the endogenous UBP-1 locus and correct excision following Rap treatment was confirmed by PCR (Fig. 5*B*). Parasites were treated with either Rap or a dimethyl sulfoxide (DMSO) control at 4 h post-invasion, and growth was followed for 7 days.

For the first cycle following Rap treatment, no growth defect was observed, possibly because of the residual presence of UBP-1 protein given its high expression at early stages. Parasites egressed normally and formed an equal number of ring stage parasites. In the second cycle after Rap treatment, the mutant parasite lines appeared to stall at the ring stage, with none progressing to form schizonts (Fig. 5*C*). This defect was confirmed by measuring the intensity of the SYBR Green DNA stain by flow cytometry of day 4 (end of second cycle post-treatment) cultures. Whereas DMSO-treated parasites contain high DNA signal because of the division into multiple merozoites in the schizont stage, the Rap-treated parasites remain in the ring stage with lower DNA content. The stalled ring-stage parasites persisted in the culture for a few days, with an increasing number of deformed or “pyknotic” parasites forming over time (Fig. 5*E*). After 3 weeks, parasites reappeared in the culture, consistent with Rap-induced excision being reversible in <1% of the population. PCR confirmed that the regrowth parasites contained the wildtype locus (Fig. 5*B*).

### *Pf*UBP-1 has wide specificity for Ub linkages

**Figure 6:**
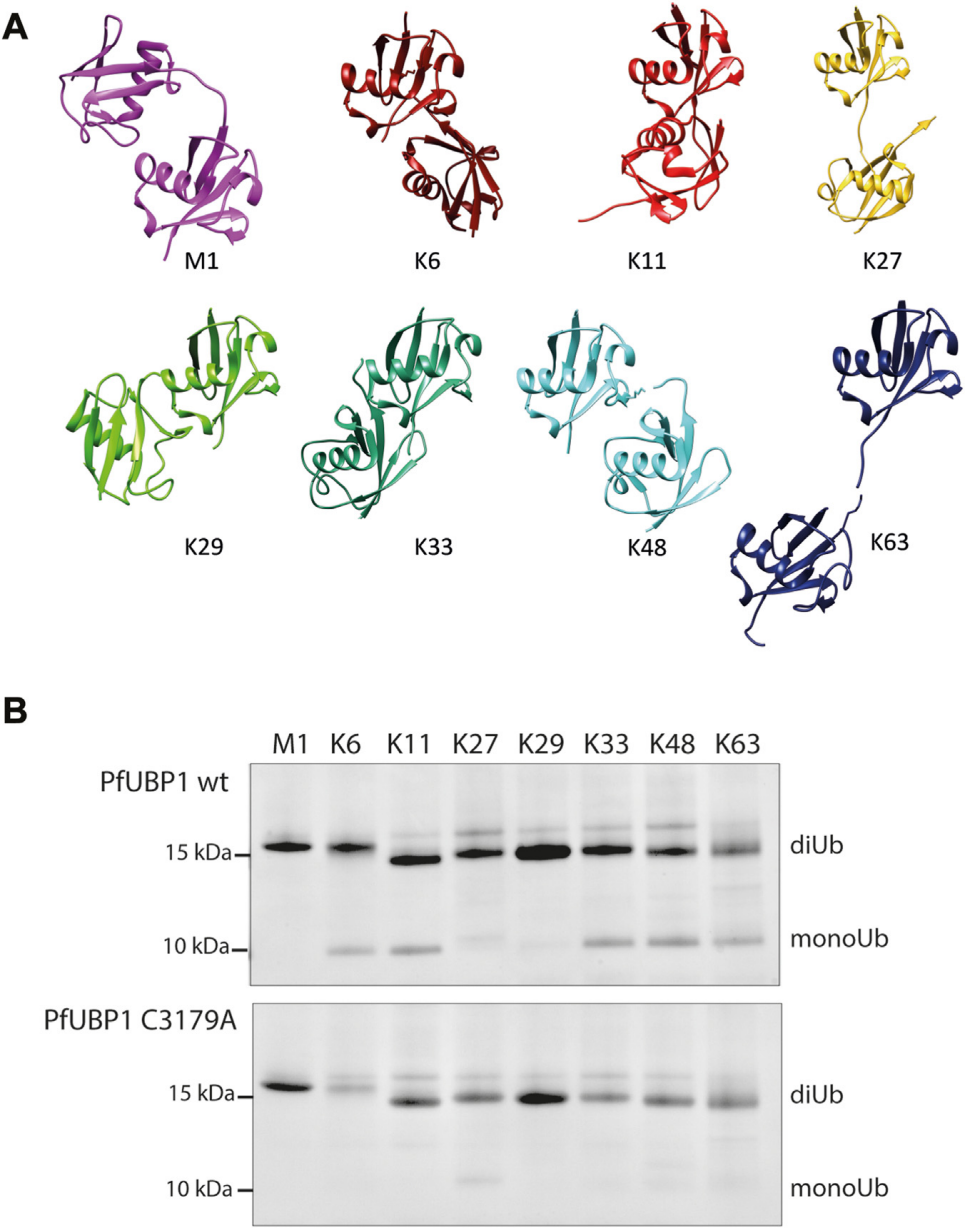
***Pf*UBP-1 linkage specificity.***A*, the panel of diUb linkage isotypes used to assess specificity. Positions of attachment are depicted by each numbered lysine. *B*, a truncation of the C-terminal end of the enzyme containing the UCH catalytic domain was expressed and mixed with each diUb individually. Activity was assessed by the release of monomeric Ub. *C* and *D*, an image of the uncropped gels can be found in Fig. S2. *Pf*UBP-1, *Plasmodium falciparum* UBP-1; Ub, ubiquitin.

Although mammalian UCH-DUBs are known to prefer cleaving Ub from small peptide fragments rather than cleaving polyUb (or diUb) chains, we wanted to assess whether this holds true for the *Pf*UBPtrunc (10, 11, 12). DUB activity of UBPtrunc was assessed by testing its ability to hydrolyze a panel of diUb linkage variants (Fig. 6*A*) (13). These variants were made using the mammalian Ub sequence, which differs in one amino acid (D16E) with *Plasmodium* Ub; however, we have previously shown that this single substitution does not affect recognition by *Plasmodium* enzymes (14, 15). UBPtrunc was incubated with each of the seven isopeptide-linked diUb variants as well as the linear M1-linked diUb followed by SDS-PAGE analysis and Coomassie Blue staining to visualize UBPtrunc-mediated proteolysis of the dimeric Ub into monomeric Ub. Apart from confirming the activity of the UBPtrunc toward dimeric Ub chains, which might reflect the recognition of longer poly-Ub chains, the results are also indicative of specificity for a particular subset of linkage types. The results showed that although *Pf*UBPtrunc cannot process linear Ub chains (M1) and at most only marginally proteolyzes K27 and K29 diUb, it is indeed a *bona fide* deubiquitinating enzyme with a wide acceptance of isopeptide linkages, able to cleave K6-, K11-, K33-, K48-, and K63-linked diUb efficiently (Fig. 6*B*). Confirming that diUb proteolytic function is also dependent on the canonical Cys-His-Asp triad, the C3179A mutant showed complete loss of function for all diUb isotypes.

## Discussion

In this study, we demonstrate the DUB activity of *Pf*UBP-1 and its ability to hydrolyze a wide range of Ub linkages. We show that point mutations associated with drug resistance markedly interfere with enzyme activity *in vitro* and, in molecular modeling *in silico*, are predicted to disrupt the stability of the catalytic domain. Despite this, these mutant *P. falciparum* lines displayed no measurable growth defect for the parasite. We confirm that complete ablation of the DUB activity is fatal for blood-stage growth in the cycle following the conditional mutation of the active site cysteine, resulting in parasites stalled at the ring stage. The lack of growth defect in parasite lines harboring either of the *Pf*UBP-1 mutants V3275F and V3306F was therefore unexpected and suggests that perhaps the low level of DUB activity retained by these drug-resistant mutants is sufficient to fulfill the essential functions of UBP-1, thus allowing the parasite to grow normally. Alternatively, the presence of the entirety of the PfUBP-1 protein may somehow correct for subtle conformational changes making the point mutations less detrimental. As the V3275F and V3306F mutants were grown in culture for some time before the growth assay was conducted, they may have compensated for the severely reduced UBP-1 activity by adjusting the expression levels of its substrates or upstream Ub ligases. An R3138H mutation of UBP-1 was previously shown to reduce parasite fitness (16); it is therefore surprising that this mutation has appeared in a small population of parasites in Asia, whereas the Val to Phe mutations have not been observed to occur naturally. It is possible that these mutations confer a stronger defect in sexual, mosquito, or liver stages of the parasite life cycle.

We and others have linked the role of *Pf*UBP-1 to reduced susceptibility to artemisinin and endocytosis in the early ring-stage parasite (4, 5, 17). UBP-1 has been localized to the cytostome by microscopy, and mislocalization of the protein results in disrupted delivery of host hemoglobin-rich cell cytosol to the food vacuole (5). In turn, reduced hemoglobin uptake has been linked to reduced drug susceptibility because of reduced artemisinin activation by heme, the degradation product of hemoglobin (18). Our results demonstrate that *Pf*UBP-1 Val to Phe mutations adjacent to the catalytic site may severely reduce DUB function, and previous results suggest parasites expressing the V3275F mutation are viable and artemisinin resistant in *P. falciparum*, whereas both Val to Phe mutations confer resistance to artemisinin in *P. berghei* and *P. chabaudi* (3, 19). The fact that both Val to Phe mutants retained some residual activity to the HA-Ub–Prg probe *in vitro* combined with our modeling results would suggest that these mutations may serve to disrupt UBP-1 interaction with the Ub target, rather than ablating proteolytic efficiency. It is possible that by slowing down UBP-1-mediated endocytic processes, the parasites are able to survive long enough to outlast the short half-life of artemisinin and complete the asexual cycle. Mutations in UBP-1 also confer resistance to chloroquine, mefloquine, lumefantrine, and piperaquine in rodent models (2, 3, 19, 20), suggesting a broader role for UBP-1. In *Plasmodium yoelii*, mutation of UBP-1 increased the ubiquitination of several substrates including multi-drug resistance protein 1 (MDR1), resulting in its mislocalization from the food vacuole to the parasite plasma membrane (20). Whether this activity is conserved in *P. falciparum* is yet to be elucidated, but defining the substrates of the deubiquitinating domain will be important for understanding its function and the implications for drug resistance.

The stalling of our conditionally inactivated UBP-1 parasites at the ring stage suggests that the *Pf*UBP-1 DUB activity, and Ub signaling in general, plays a central role in host cytosol uptake and delivery to the food vacuole to sustain parasite development. DUB involvement in endocytosis has been described in higher eukaryotes, although these processes are notably distinct from the host cytosol ingestion of *Plasmodium* and, further, UBP-1 bears no resemblance to AMSH and UBPY, the mammalian enzymes involved (21). Of note, the Ub linkage preference profile of UBP-1 seems identical to that of UBPY (USP8) and more closely resembles that of other USP-type DUBs (22). Although UBP-1 is predicted to have a UCH domain, it is possible that it interacts with Ub in a manner more reminiscent of USPs (23). Further structural studies are needed to understand how it folds and its exact mode of binding to Ub. The upstream region of UBP-1 also contains a VHS domain often present in endocytic proteins, which has not been previously observed in a DUB enzyme (24). Other proteins connected to the Ub pathway have also been localized to the cytostome, including EPS15, which in higher eukaryotes is responsible for recruiting ubiquitinated membrane receptors to the endocytic machinery. Kelch13, a cytostome component strongly associated with artemisinin resistance, also resembles Ub ligase substrate adaptors (25). Defining how ubiquitination and deubiquitination mediates the divergent endocytic machinery of the parasite could inform the design of artemisinin combination therapies to combat drug resistance.

The role of Ub signaling for protein trafficking rather than for protein degradation is not well understood and has not been investigated in *P. falciparum*. Despite the evolutionary conservation of the Ub–proteasome system across eukaryotes, few predicted that *Plasmodium* DUBs appear to be direct orthologs of characterized enzymes in other organisms (15, 26). The unusual biology of *Plasmodium* undoubtedly requires the action of specialized proteins capable of supporting its unique growth and development. Defining their behavior, both temporally and functionally, will undoubtedly uncover a number of novel targets for antimalarial development and increase our understanding of cellular homeostasis in *Plasmodium* parasites.

## Experimental procedures

### Cloning and mutagenesis for *Escherichia coli* expression

The C-terminal region of PfUBP-1 (PF3D7_0104300) spanning amino acids 2952 to 3499 and containing the catalytic C19 peptidase domain was amplified from *P. falciparum* 3D7 complementary DNA (forward primer: 5′-TATGGATCCCATATGATGAAAAATGTAAAAC-3′ and reverse primer: 5′-TCTAAAGCTTTTAAAAGTACAAATCTGGAG-3′) and cloned into a SUMO-modified pet28a vector (kind gift from Dr Owain Bryant) to yield a protein with a 6xHis and SUMO tag on the N terminus. Point mutations C3179A, V3275F, and V3306F were introduced using the QuickChange II Site-Directed Mutagenesis Kit (Agilent).

### Protein expression and enrichment

SoluBL21 *E. coli* (Genlantis) were used for expression of wildtype and mutant *Pf*UBP-1 protein variants. Bacterial cultures were grown at 37 °C, and protein expression was induced with 0.16 mM IPTG at an absorbance of 0.6 at 600 nm, then grown for 16 to 20 h at 18 °C. Protein was enriched under native conditions according to the QIAexpressionist (Qiagen) protocol. Bacterial pellets (1 g for each protein) were resuspended in lysis buffer (50 mM NaH_2_PO_4_, 300 mM NaCl, 10 mM imidazole, 1 mM DTT, 5 U/ml benzonase, 2 μg/ml aprotinin, pH8) and passed through a cell disruptor at 30 kpsi. The lysates were centrifuged at 40,000*g* for 1 h at 4 °C. The supernatants were mixed with 50 μl CoNTA beads (Generon) and incubated for 2 h at 4 °C on a rotary shaker. After the incubation, the beads were centrifuged, the flow-through was removed, and the beads were washed six times in wash buffer (50 mM NaH_2_PO_4_, 300 mM NaCl, 40 mM imidazole, pH 8). As the attempts to elute *Pf*UBP-1 from the beads yielded inactive protein, all subsequent reactions were performed with *Pf*UBP-1 on beads. Although the protein was not detectable by Coomassie because of low expression levels (Fig. S2*B*), anti-His Western blots confirmed that a protein of the correct size was observed (Fig. 4*B*). As such, concentrations were calculated by eluting bound protein from a defined volume of beads and quantifying by Western blot. Normalized volumes of beads bound to defined amounts of protein were then used in each assay.

### DiUb synthesis

All isopeptide-linked diUb chains were generated using total chemical protein synthesis of monoUb mutants in analogy to the described procedure by El Oualid *et al.* (27). In short, a Ub mutant equipped with a γ-thiolysine on the prospective lysine was reacted with a Ub–thioester in denaturing buffer (8 M guanidium–HCl/100 mM phosphate buffer at pH 7.6 supplemented with 100 mM Tris(2-carboxy-ethyl)phosphine) and 100 mM 4-mercaptophenylacetic acid. The crude diUb was first purified using reversed-phase HPLC purification and subsequently desulphurized using 2,2′-Azobis[2-(2-imidazolin-2-yl)propane] Dihydrochloride (VA044) and reduced glutathione in 8 M guanidium–HCl/100 mM phosphate buffer with 100 mM Tris(2-carboxy-ethyl)phosphine at pH 7.0. Purification using reversed-phase HPLC purification and S75 16/600 Sephadex size-exclusion column (GE Healthcare) chromatography using 20 mM Tris, 150 mM NaCl at pH 7.6 resulted in the natively isopeptide-linked diUb that was concentrated by spin filtration and snap frozen and stored at −80 C until further use. M1-linked diUb was recombinantly expressed following the procedure earlier reported by Rohaim *et al.* (28). All diUbs are based on the human Ub sequence, which deviates at position 16 from the parasite sequence (human: E, *Plasmodium falciparum*: D). This however has been shown to not impact recognition by plasmodium DUBs.

### DiUb cleavage assay

*Pf*UBP-1 wildtype or the catalytically inactive *Pf*UBP-1 C3179A mutant (approximately 50 ng in a 10 μl CoNTA bead slurry) were incubated with 2 μg diUb in reaction buffer (50 mM NaH_2_PO_4_, 300 mM NaCl, and 1 mM DTT) for 1 h at 37 °C. The reactions were terminated by adding 4× reducing sample buffer (Boston Biochem) supplemented with 100 mM DTT and loaded on gel for SDS-PAGE analysis. Visualization of the protein bands was performed using Coomassie Blue staining.

### Activity-based probe assay

PfUBP-1 wildtype and mutant proteins (approximately 50 ng in a 10 μl CoNTA bead slurry) were incubated with 10 μg of Ub–Prg probe in reaction buffer (50 mM NaH_2_PO_4_, 300 mM NaCl, and 1 mM DTT) for 1 h at 37 °C. The reactions were terminated by adding 4× reducing sample buffer (Boston Biochem) supplemented with 100 mM DTT.

### AMC assay

AMC assays were performed as described previously (26). Briefly, the reactions were performed in AMC buffer (50 mM Tris [pH 7.4], 150 mM NaCl, 2 mM EDTA, 2 mM DTT, and 1 mg/ml bovine serum albumin). The reactions were initiated by the addition of 250 nM Ub–AMC (Boston Biochem) substrate to PfUBP-1 wildtype or mutant proteins (approximately 50 ng in a 10 μl CoNTA bead slurry). The fluorescence was measured on a BMG FLUOstar Omega plate reader continuously for 1 to 3 h at room temperature using an excitation wavelength of 355 nm and an emission wavelength of 460 nm, with shaking before each timepoint.

### Parasite growth

*P. falciparum* 3D7 parasites were grown in RPMI1640 with 50 mg/l hypoxanthine, 0.25% sodium bicarbonate, 0.5% Albumax II, 25 mM Hepes (Life Technologies), supplemented with 1× concentration (2 mM) GlutaMAX (Gibco) and 25 μg/ml gentamicin (Melford Laboratories). Human red blood cells were used for propagation of *P. falciparum* 3D7 parasites at 4% hematocrit.

### Competition assays

Growth of mutant parasites was directly assessed against a Dd2-EGFP reference line as previously described (29). Briefly, equal numbers of unmarked wildtype (3D7), V3275F, and V3306F parasites were mixed with Dd2-EGFP and assessed for growth over time using flow cytometry. Mixtures were stained with MitoTracker Deep Red to detect all parasites, and ratios of nonfluorescent test line to fluorescent reference line were measured to calculate growth relative to the reference line.

### Plasmid generation and parasite transfection

For the straight mutation of the active site cysteine to alanine (C3179A), CRISPR–Cas9 was used to target directly upstream of the active site using one of two different guide sequences (guide 11: AAATTCATTAATCCTACAGGTGG or guide 13: AATTATCCACATCCACCTGTAGG) within a pDC2 plasmid containing Cas9, tracR RNA, and an hDHFR cassette for WR99210 resistance. The homology repair sequence was also inserted into the Cas9 plasmid and included 5′ and 3′ homology regions flanking a 40 bp recodonized sequence encompassing the guide sequence and the C > A mutation. A C > C control plasmid with no mutation was also included and transfected at the same time with the same parasite batch, as a control. Parasites were cultured for a maximum of 2 months until it could be ascertained whether transfection was successful, and if so, the locus was PCR amplified and sequenced by Sanger sequencing (Genewiz).

For the conditional C3179A mutation, the same guide (#11) as for the straight mutation was used to target the endogenous UBP-1 locus. The repair plasmid contained a pMK-RQ backbone (Integrated DNA Technologies) and Gibson-assembled fragments: a 536 bp 5′ homology region, first loxP intron, recodonized UBP-1 UCH domain with wildtype cysteine, second loxP intron, recodonized UBP-1 UCH domain with cysteine to alanine mutation, and a 305 bp 3′ homology region. The wildtype and mutant recodonized regions were differently recodonized to prevent spontaneous homologous recombination using the “codon optimization tool” from Integrated DNA Technologies, and synthesized by ThermoFisher. No tags were included on UBP-1 because of difficulty obtaining parasites using a tagged version of the plasmid.

For each transfection, 60 μg of the repair plasmid and 20 μg of the Cas9 plasmid were transfected into 3D7 DiCre schizont-stage parasites using the P3 Nucleofector kit (Lonza) and selected with 2.5 nM WR99210 between days 1 and 10. Treatment with 20 nM Rap induced the DiCre-mediated excision of the wildtype DUB domain and brought the C3179A mutant domain into frame with the N terminus of the gene. Correct integration into the genome and excision upon Rap treatment were confirmed by PCR.

### Measuring growth of conditional mutant parasites by flow cytometry

To measure the growth of UBP-1 conditional mutant parasites, early ring-stage parasites were treated with 20 nM Rap or a DMSO control overnight. From 24 h post-treatment, an aliquot of both cultures was taken daily and stained with SYBR Green DNA stain. Parasitemia was measured using an Attune NxT flow cytometer with a 488 nm (blue) excitation laser, a 505 nm longpass filter, and a 530/30 nm bandpass filter. Giemsa smears were taken daily to observe parasite morphology. Experiments were repeated in biological triplicate.

### Immunoblot

Protein levels were normalized using bicinchoninic acid (Pierce) and separated by SDS-PAGE before semidry transfer to polyvinylidene difluoride at constant 140 mA for 1 h. Membranes were blocked in 5% milk and probed with 3F10-horseradish peroxidase anti-HA antibody (Roche) followed by imaging with enhanced chemiluminescence.

### Structural modeling

A molecular model of the C terminus of *Pf*UBP-1 (2952-3499) was generated by AlphaFold2 (30). The molecular consequences of the V3275F and V3306F were analyzed for their effects on the protein structure (31). The effects of the mutations on the stability of *Pf*UBP-1 were predicted using mCSM-Stability (32), DUET (33) and SDM (34). The effects of the mutations on protein stability, dynamics, and flexibility were assessed using DynaMut (35) and DynaMut2 (36). The molecular interactions made by the wildtype and mutant residues were calculated and visualized using Arpeggio (37).

## Data availability

All data described are available within the article.

## Supporting information

This article contains supporting information (Figs. S1 and S2).

## Conflict of interest

The authors declare that they have no conflicts of interest with the contents of this article.
