# Supplementary material for: Drug resistance–associated mutations in Plasmodium UBP-1 disrupt its essential deubiquitinating activity
Source: J Biol Chem. 2025 Feb 3;301(3):108266. doi: 10.1016/j.jbc.2025.108266 (PMC11927682; doi:10.1016/j.jbc.2025.108266)
Supplement: Supplementary Figure 1 [file mmc1.pdf]

a

	C>A	C>C
Transfection 1 (guide #11)	Successful	Failed - no parasites recovered
Transfection 2 (guide #13)	Successful	Failed - Cysteine not mutated
Transfection 3 (guide #11)	Successful	Failed - wild-type parasites recovered

b

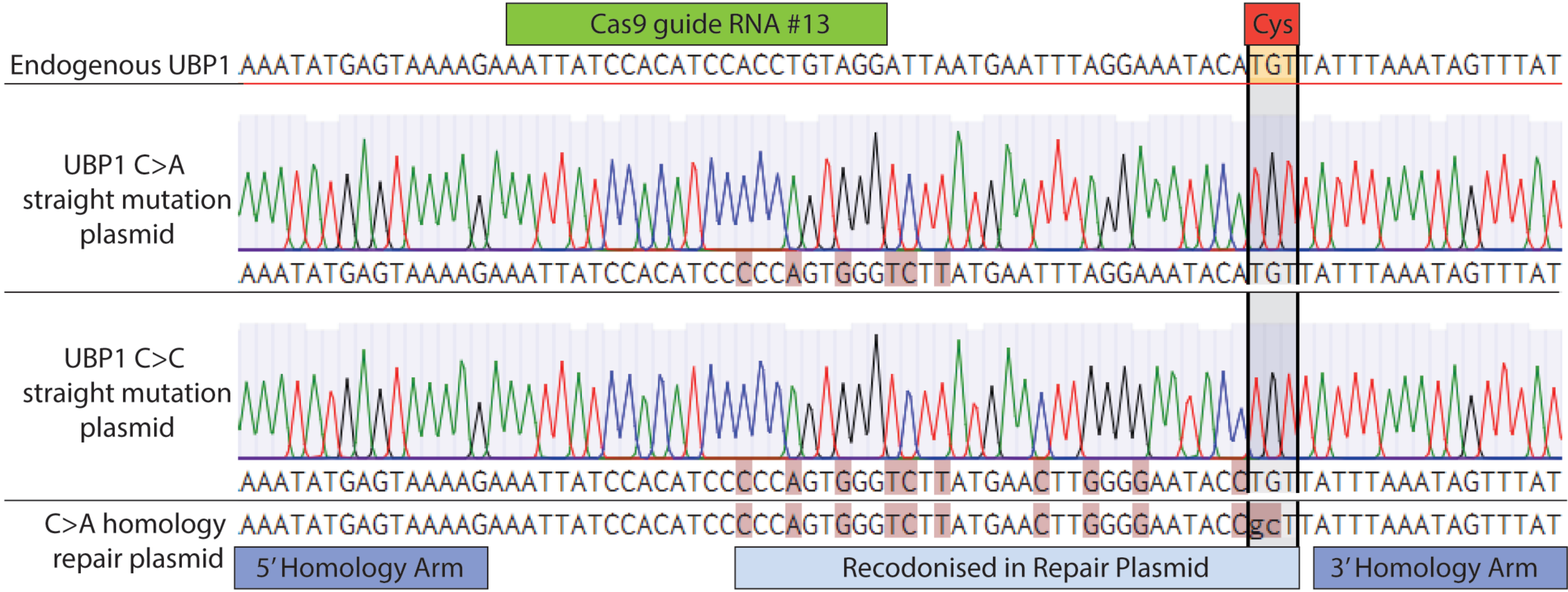

Supplementary Figure 1. (A) Table of results of transfections for the straight mutation of UBP1 C>A. All three C>C control mutations generated modified parasites while the C>A mutant transfections were not successful. (B) Sequencing trace for one failed C>A transfection where the guide sequence was successfully mutated but not the C>A site.
