# Supplementary material for: Drug resistance–associated mutations in Plasmodium UBP-1 disrupt its essential deubiquitinating activity
Source: J Biol Chem. 2025 Feb 3;301(3):108266. doi: 10.1016/j.jbc.2025.108266 (PMC11927682; doi:10.1016/j.jbc.2025.108266)
Supplement: Supplementary Figure 2 [file mmc2.pdf]

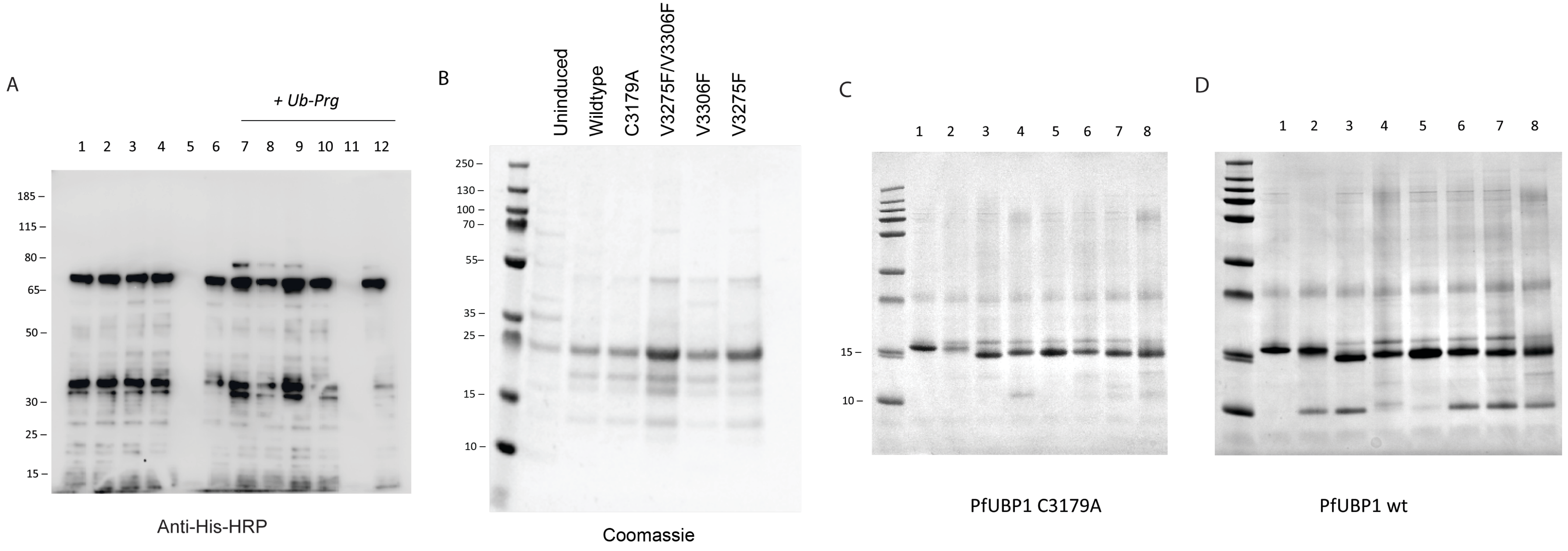

Supplementary Figure 2. Uncropped western blots and Coomassie gels. (A) Uncropped western blot of recombinant UBP1 constructs before and after the addition of Ub-Prg (from figure 4B). (B) Coomassie gel of recombinant UBP1 eluted from beads. UBP1 itself is not detectable at this level. (C) and (D) Uncropped gels from figure 6B, representing cleavage of di-Ub.
